# Widespread Inter- and Intra-Domain Horizontal Gene Transfer of d-Amino Acid Metabolism Enzymes in Eukaryotes

**DOI:** 10.3389/fmicb.2016.02001

**Published:** 2016-12-20

**Authors:** Miguel A. Naranjo-Ortíz, Matthias Brock, Sascha Brunke, Bernhard Hube, Marina Marcet-Houben, Toni Gabaldón

**Affiliations:** ^1^Centre for Genomic Regulation, The Barcelona Institute of Science and TechnologyBarcelona, Spain; ^2^Universitat Pompeu FabraBarcelona, Spain; ^3^Fungal Genetics and Biology Group, School of Life Sciences, University of NottinghamNottingham, UK; ^4^Department of Microbial Pathogenicity Mechanisms, Hans Knoell Institute JenaJena, Germany; ^5^Friedrich Schiller UniversityJena, Germany; ^6^Center for Sepsis Control and Care, University HospitalJena, Germany; ^7^Institució Catalana de Recerca i Estudis Avançats (ICREA)Barcelona, Spain

**Keywords:** horizontal gene transfer, d-Amino acid metabolism, amino acid racemase, d-Amino acid oxidase, *Candida glabrata*, fungi, Abaccus

## Abstract

Analysis of the growing number of available fully-sequenced genomes has shown that Horizontal Gene Transfer (HGT) in eukaryotes is more common than previously thought. It has been proposed that genes with certain functions may be more prone to HGT than others, but we still have a very poor understanding of the selective forces driving eukaryotic HGT. Recent work uncovered that d-amino acid racemases have been commonly transferred from bacteria to fungi, but their role in the receiving organisms is currently unknown. Here, we set out to assess whether d-amino acid racemases are commonly transferred to and between eukaryotic groups. For this we performed a global survey that used a novel automated phylogeny-based HGT-detection algorithm (Abaccus). Our results revealed that at least 7.0% of the total eukaryotic racemase repertoire is the result of inter- or intra-domain HGT. These transfers are significantly enriched in plant-associated fungi. For these, we hypothesize a possible role for the acquired racemases allowing to exploit minoritary nitrogen sources in plant biomass, a nitrogen-poor environment. Finally, we performed experiments on a transferred aspartate-glutamate racemase in the fungal human pathogen *Candida glabrata*, which however revealed no obvious biological role.

## Introduction

Horizontal Gene Transfer (HGT) refers to the movement of genetic material between two organisms for which a significant reproductive barrier exists (Doolittle, 1999). In bacteria and archaea, HGT is widely acknowledged as a powerful evolutionary force that shapes a large fraction of the genome, expands the scope of a species' pangenome, and drives adaptation to new niches (Soucy et al., 2015). In contrast, eukaryotic HGT was long thought to be extremely rare, and limited to few microbial taxa (Eisen, 2000; Andersson, 2005). However, in recent years, the number of described events of eukaryotic HGT has increased exponentially, suggesting it is more widespread than previously anticipated (Andersson, 2005, 2009; Keeling and Palmer, 2008; Laura, 2015; Soucy et al., 2015). However, we still have, a very limited understanding of the mechanisms that underlie the transfer of genetic material in eukaryotes. Many possible mechanisms have been put forward, including acquisition of genetic material through direct phagocytosis, transformation, or mediation by intracellular bacteria, organelles, symbionts, parasites, viruses, or plasmids (Doolittle, 1999; Huang, 2013; Gluck-Thaler et al., 2015; Lacroix and Citovsky, 2016). These different mechanisms have been attributed to different taxonomic groups depending on their life styles, but most remain without a direct empirical demonstration of their role in mediating HGT. For instance the formation of resistance forms such as spores, cysts, or dehydration-induced cryptobiosis, and the associated DNA repair mechanisms may facilitate acquisition of foreign DNA, as it has been proposed for the bdelloid rotifer *Adineta vaga* (Flot et al., 2013). Similarly, phagocytotic microbes may acquire genes from their prey, as proposed by the famous “you are what you eat hypothesis” (Andersson, 2005, 2009). In fungi, which possess a cell wall and cannot perform phagocytosis, the above mentioned mechanisms seem implausible. However, in this group cell fusion may lead to the formation of, perhaps transient, heterokaryotic lines, providing an opportunity for HGT (Roper et al., 2011, 2013). Finally, HGT mediated by bacterial endosymbionts, parasites, or viruses may affect all eukaryotic groups (Andersson, 2009; Wijayawardena et al., 2013).

Regardless of the underlying mechanism, HGT represents a form of non-vertical (or reticulate) evolution which results in phylogenetic incongruence between gene and species evolutionary histories. Hence, most HGT detection methods rely on the detection of such incongruences through the analysis of similarity profiles or gene phylogenies and their comparison to taxonomy or species phylogenies, respectively (Whitaker et al., 2009a,b; Leigh et al., 2011). However, inference of HGT in the presence of phylogenetic incongruence is far from straightforward. First, phylogenetic incongruence can be caused by many other factors not related to HGT, such as differential gene duplication and loss, lineage-specific accelerated evolution, or compositional biases (Galtier and Daubin, 2008). This problem is exacerbated at short evolutionary distances where the phylogenetic incongruence introduced by HGT is small, and comparable to that caused by other sources; or at very large distances, where the phylogenetic signal may not be strong enough to robustly resolve the evolutionary history of the putatively transferred gene. Second, phylogenetic information may be limited in terms of existing or available species; or controversial, with different trees resulting in conflicting species relationships. Third, phylogenetic reconstruction or similarity searches rely on sequence data, which is not free of errors. Indeed, certain biological samples are, by nature, prone to contamination. For example, it is highly difficult to separate genomic DNA of the host from internal parasites or commensals (Wijayawardena et al., 2013; Grant and Katz, 2014). In other cases, incomplete genome assemblies or annotations may result in apparent gene losses. Finally, incomplete sampling of extant species (or genes), the inaccessibility of extinct lineages, the possibility of multiple transfer events, extensive sequence divergence, and other factors are significant impediments (phylogenetic artifacts, limited robustness) for reconstructing past HGT events (Gluck-Thaler et al., 2015; Soucy et al., 2015). Thus, any HGT prediction method is prone to report many false positives, which has to be filtered out through manual curation. In the absence of accurate models of genome evolution, there is currently no universally applicable statistical framework that allows assessing the significance of HGT prediction. As a consequence, criteria may vary widely across studies, which complicates comparisons.

It is currently unknown whether certain families of proteins are particularly prone to HGT in eukaryotes. Previous studies, however, have found several independent events involving a given protein family or proteins from related pathways, suggesting that proteins encoding certain functions may be prone to HGT. This is the case for amino acid racemases, which have been transferred at least eight independent times from bacteria to fungi, according to a phylogenomic analysis of 60 fully-sequenced fungal genomes (Marcet-Houben and Gabaldón, 2010). This suggests a high frequency of HGT for this kind of enzymes in fungi, but it is unclear whether such transfers have occurred in other eukaryotic groups. Moreover, that study particularly focused on transfers from bacteria to fungi, but potential secondary transfers among fungi, or between fungi and other eukaryotes were not assessed.

Amino acid racemases catalyze the interconversion of enantiomers of biological molecules. The best known of these molecules are amino acids, and racemases catalyze the change from the proteinogenic l-amino acids to their d-amino acids counterparts and vice versa (Yoshimura and Esak, 2003; Friedman and Island, 2010). These enzymes can be further divided into two main classes: Pyridoxal phosphate (PLP)-independent enzymes, that act on acid amino acids (glutamate and aspartate), hydantoin and related compounds; and PLP-dependent enzymes, that act on most other amino acids, and often have affinity for more than one substrate (Seebeck and Hilvert, 2003; Yoshimura and Esak, 2003). Each class contains several distinct families. Other enzymes acting on d-amino acids include d-amino acid oxidases, that remove the amino group releasing free ammonia and a carboxylic acid backbone (Pollegioni et al., 2007; Friedman and Island, 2010); and d-alanine-d-alanine ligases, ATP-dependent enzymes that catalyze the binding of two d-alanine molecules during the biosynthesis of peptidoglycan in bacteria (Hrast et al., 2012).

Amino acid racemases have been widely studied due to their important role in the biosynthesis of peptidoglycans and some other bacterial polymers (Yoshimura and Esak, 2003; Friedman and Island, 2010). They are also well-known players in the central nervous system, as some of them are neuroactive or act as biosynthetic precursors of neuroactive compounds (D'Aniello, 2007; Wolosker et al., 2008; Ohide et al., 2011). d-amino acids are substrates for the synthesis of some complex secondary metabolites, such as many non-ribosomal peptides (von Döhren et al., 1999; Schwarzer et al., 2003; Marahiel, 2009; Strieker et al., 2010; Hur et al., 2012) or alkaloids (Mootz and Marahiel, 1997) that are produced by many eukaryotes such as fungi or plants. Other described but less studied roles of d-amino acids include developmental regulation in certain groups of vertebrates (D'Aniello, 2007; Wolosker et al., 2008; Canu et al., 2014), osmoprotection and development in some invertebrates (Abe et al., 2005; Yoshikawa et al., 2011), regulation of biofilm production in several bacterial species (Cava et al., 2011), and calcium channel regulation in pollen tubes in *Arabidopsis* (Michard et al., 2011). d-amino acids can also accumulate in soils and aquatic sediments under the right conditions, as a result of both the spontaneous racemization of free amino acids or the accumulation of peptidoglycan and other d-amino acid rich bacterial polymers (Vranova et al., 2011; Steen et al., 2013; Zhang and Sun, 2014). Hence, d-amino acids may constitute a relevant nutrient source for soil microbes, although this aspect has not been comprehensively studied. Lastly, aspartate has a higher spontaneous racemization rate than other amino acids and thus d-aspartate tends to accumulate in damaged or aged proteins (Fujii et al., 2011; Ohide et al., 2011). Moreover, amino acid racemases are widespread across eukaryotic genomes, and several clades of eukaryotic racemases are known whose function is poorly understood (Yoshimura et al., 1996; Yoshimura and Esak, 2003; Uo et al., 2001; Fujitani et al., 2007; Uda et al., 2015). All of this suggests that there may exist other functions of d-amino acids beyond the ones listed above.

d-amino acid metabolic genes have been transferred to eukaryotes several times independently (Marcet-Houben and Gabaldón, 2010), this must imply that these organisms have been exposed to environments where the ability to degrade d-amino acids is advantageous. Catabolism of d-amino acid may be important in environments where other nitrogen sources are scarce. d-amino acids are considerably toxic for many organisms, including many bacteria, yeasts and plants (Yow et al., 2006; Chen et al., 2010; Gördes et al., 2011, 2013; Zhang and Sun, 2014; Leiman et al., 2015), and thus the ecological interactions between fungi, plants and environmental d-amino acids is worth examining. At least two pathogenic yeast species, *Candida glabrata* and *Candida orthopsilosis* harbor their own horizontally acquired amino acid racemases (Fitzpatrick et al., 2008; Marcet-Houben and Gabaldón, 2010), an aspartate-glutamate-hydantoin racemase and a proline racemase, respectively. Little is known about the role of these enzymes in *Candida*. Better characterized is a transferred proline racemase in some *Trypanosoma* species (Chamond et al., 2009). Functional studies have proved that the introduction of d-proline residues in surface proteins greatly reduces the immune response toward the parasite (Chamond et al., 2009; Coutinho et al., 2009). An additional HGT event of an alanine racemase in *Adineta vaga* has been reported (Flot et al., 2013), although *Adineta* was not included in this study.

Here, we set out to assess the extent of HGT affecting d-amino acid metabolism in Eukaryotes. For this we performed a global phylogenomics survey, and developed a novel automated phylogeny-based HGT-detection algorithm (Abaccus). This automated pipeline, followed by manual curation of the HGT candidate events revealed 150 eukaryotic racemases resulting from HGT, which represents 7.0% of the eukaryotic racemase repertoire. Even more, we were able to detect a significative number of HGT events between pairs of eukaryotic lineages. Given that this kind of events is much more difficult to detect than HGT between prokaryotes and eukaryotes, our data suggest that inter-eukaryotic transfer must be fairly common. Detected transfers involved several eukaryotic groups, and several classes of amino acid racemases, but where not similarly distributed in the different groups. Among fungi, we found a significant enrichment of HGT-derived racemases in plant-associated fungi. Based on this, we hypothesize for this group a possible role of d-amino acid racemases in the utilization of d-amino acids as nitrogen source. Finally, in an attempt to characterize a transferred racemase gene, we performed experiments on CAGL0D01210g, a bacterial-derived aspartate-glutamate racemase from *C. glabrata*. CAGL0D01210g was detected as an HGT event by Marcet-Houben and Gabaldón (2010). Its sequence has ~60% identity with aspartate racemase genes in *Lactobacillus* sp. and other bacteria; while no similar sequence can be found in other yeast species. Our results provide a global survey of racemase HGT in fungi and set the ground for more extensive studies of eukaryotic HGT.

## Materials and methods

### Sequence data

All protein sequences were downloaded from the Uniprot database (The Uniprot Consortium, 2014). The eukaryotic complete proteomes were downloaded from the pool of all available eukaryotic proteomes as of May 2015 (see Supplementary Table 1 for a complete list of the analyzed proteomes). This dataset included a total of 478 eukaryotic organisms and 6,842,114 protein sequences, and hereafter will be referred as euka_proteomes. The reference prokaryotic sequences were obtained from Uniref clusters of sequences (Uniprot database) as of May 2015, filtered for taxonomy equal to “Bacteria” (TaxID: 2) or equal to “Archaea” (TaxID: 2157) and identity threshold equal to 0.5. Hereafter we will refer to this dataset as proka_ref50. We used this clustered dataset in order to avoid over-representation of closely related sequences derived from multiple strains of the same species, as well as to reduce the overall size of the database, while keeping a balanced taxonomic representation.

### Sequence profile searches

Based on existing literature, we manually selected a set of d-amino acid metabolism protein domains from the Pfam database (Finn et al., 2014; Table 1). Then, for every domain in the list, we performed a search using HMMsearch (HMMer version 3.0; Finn et al., 2011) against the euka_proteomes dataset in order to retrieve all the eukaryotic proteins that contain the given domain. The results were filtered to keep only matches containing a first hit *e*-value lower than 10^−5^. Subsequently, for each previously identified sequence, we performed an iterative homology search against a combined database comprising euka_proteomes and proka_ref50 datasets using Jackhmmer (HMMer version 3.0) with two iterations and using an *e*-value cutoff of 10^−10^. In each case, the best 150 hits were selected and used for the phylogenetic analysis described below.

**Table 1:** **Analyzed families and their corresponding Pfam domains**.

**Name**	**PfamID**	**Description**
Ala_racemase_C	PF00842	C terminal domain of PLP-dependent racemases of alanine and other non-acidic amino acids
Ala_racemase_N	PF01168	N terminal domain of PLP-dependent racemases of alanine and other non-acidic amino acids
Asp_Glu_race	PF01177	PLP-independent racemases of aspartate, glutamate and hydantoin
Pro_racemase	PF05544	PLP-independent proline and hydroxyproline racemases
Racemase_4	PF13615	Putative eukaryotic alanine racemases
DAO	PF01266	FAD-dependent oxidoreductase. Includes many members that act on d-amino acids.
Dala_Dala_lig_C	PF07478	C terminal domain of enzymes related to d-alanine d-alanine ligases involved in peptidoglycan biosynthesis. Catalytic domain.
Dala_Dala_lig_N	PF01820	N terminal domain of enzymes related to d-alanine d-alanine ligases involved in peptidoglycan biosynthesis. Substrate binding domain.

*Columns indicate, in this order, the name of the family, the Pfam ID, and the description available in the Pfam database*.

### Phylogenetic analysis

We used the PhylomeDB pipeline (Huerta-Cepas et al., 2011) to reconstruct the phylogenies of each putative selected HGT event. In brief, all the homologous sequences for each candidate were aligned bidirectionally using MAFFT v6.861b (Katoh and Standley, 2013), Kalign v2.04 (Lassmann et al., 2009), and MUSCLE v3.8 software (Edgar, 2004). The six resulting alignments were used to generate a consensus alignment with M-coffee software v10.00.r1607 (Wallace et al., 2006). Alignments were filtered using trimAl v1.3 (Capella-Gutiérrez et al., 2009), using a consistency cut-off of 0.16667 and a gap threshold of 0.1. Phylogenetic trees were then reconstructed using PhyML 3.0 (Guindon et al., 2010). In a first step neighbor joining trees were reconstructed using BioNJ as implemented in phyML, and their likelihoods were estimated by PhyML, allowing branch length optimization, for each of the following seven different evolutionary models (JTT, WAG, MtREV, VT, LG, Blosum62, CpREV, and DCMut). The best fitting model was chosen according to the AIC criterion (Akaike, 1973), and was then used to reconstruct a maximum likelihood (ML) tree. ML trees were reconstructed using PhyML. In all cases, a discrete gamma-distribution model with four rate categories plus invariant positions was used. The gamma parameter and the fraction of invariant positions were estimated from the data. Branch support was evaluated with an approximate Likelihood Ratio Test (aLRT).

### Taxonomic database

To provide an evolutionary framework to estimate a minimal number of losses and to contrast alternative scenarios (exclusively vertical evolution), we constructed a taxonomic database based on the taxonomic information provided by NCBI and the metadatabase Catalog of Life (Bisby et al., 2010) for the organisms present in the proteome database (Bisby et al., 2010). We used this approach instead of a real phylogenetic tree for a number of reasons. First, phylogenetic relationships between many of the deeper branches of the eukaryotic tree of life, as well as the phylogeny of some particular taxa, are still under debate. The possibility of generating a unified phylogenetic tree for all the organisms would be either highly expensive (phylogenomic approach) or highly inaccurate, given the fact that our dataset contained problematic taxa (e.g., highly reduced parasites) and a very uneven representation of taxa; factors that are expected to produce problematic phylogenies. Instead, we used taxonomy as an approximation to phylogenetic relationships. Since the taxonomic information available for different organisms is uneven, we manually modified the NCBI taxonomy so it contained the same number of categories across the diversity of eukaryote (species, genus, family, order, class, phylum, kingdom, superkingdom, and domain). For groups in which the taxonomy is not well-resolved in NCBI we consulted the more recent phylogenetic studies (Riisberg et al., 2009; Vossbrinck et al., 2010; Adl et al., 2012). The main objective was to provide a topologically correct and balanced taxonomy, even at the expense of losing well-known relationships between clades. The curated taxonomy used in this study is available at (Supplementary Table 1).

### Detection of HGT

**Figure 1:**
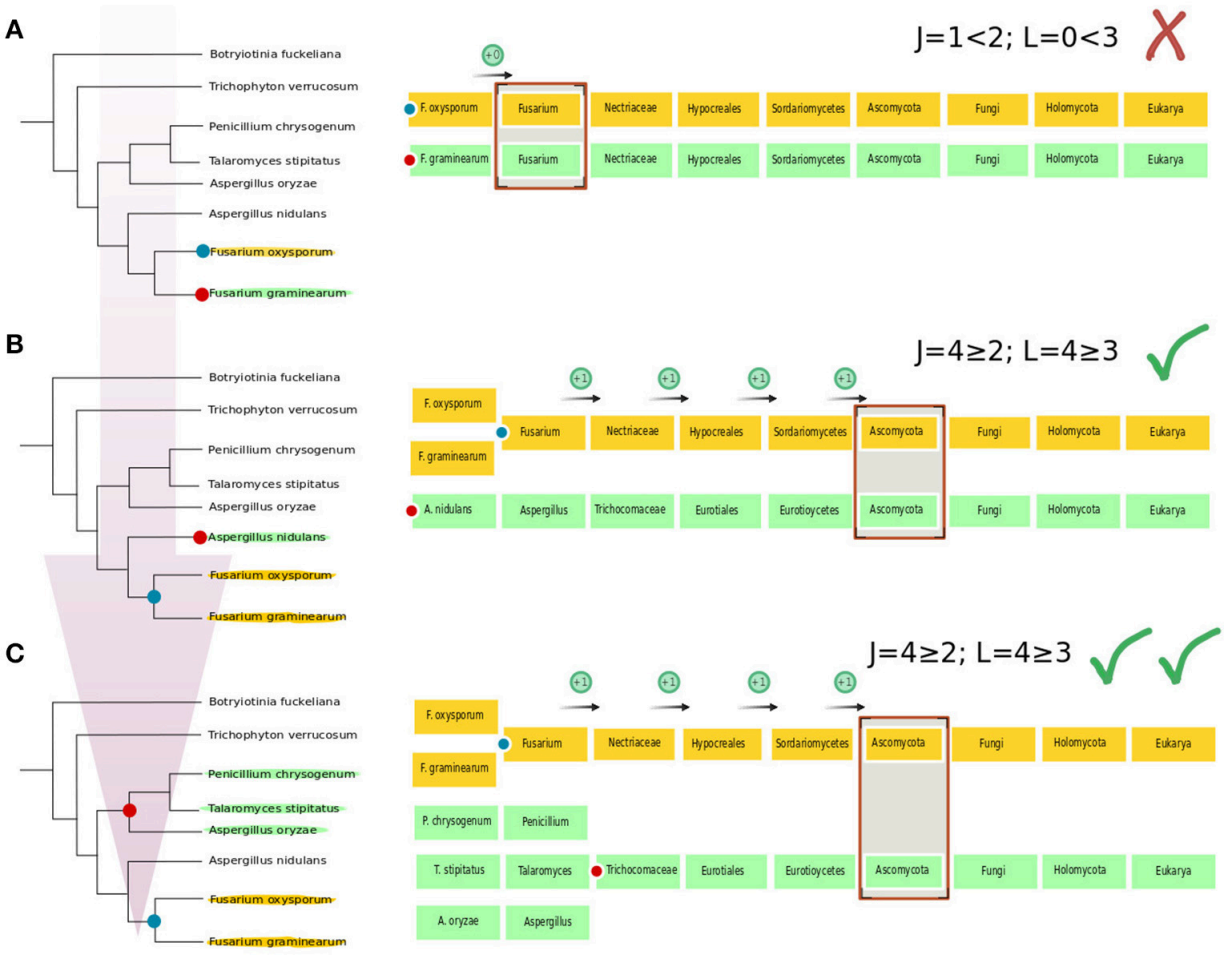
**Schematic example of the Abaccus algorithm**. We use a simple example of a tree in which a sequence from *Fusarium oxysporum* has been used as a seed. The tree node that is most distant to the seed has been set as the root. **(A)** In a first step, Abaccus progresses from the seed sequence (blue dot) toward the root and finds the sister branch of the seed sequence (red dot), in this case that node has only a descendant leaf, a sequence from *F. graminearum*. The taxonomy of the sequences contained in the current (seed) and sister nodes are compared. In this case both sequences share the genus level (*Fusarium*). The number of taxonomic levels from the current node (*Fusarium oxysporum*) to the lowest common taxonomic category (*Fusarium*) is just one (from species to genus level). Thus, parameter J = 1. Losses (L) are then established by counting how many lineages are present in the database but do not appear in the considered subtree. In this case the number of losses is L = 0. Since J and L are lower than the cutoff (J ≥ 2 and L ≥ 3), we conclude that this particular node is not the result of HGT. **(B)** Abaccus proceeds by setting the current node as the next node in direction to the root (blue dot), and establishes the sister node (red dot) in the same manner. In this case, the current node includes the sequences of both *Fusarium* species. The sister node contains a sequence from *Aspergillus nidulans*. The genus *Fusarium* and *Aspergillus nidulans* are both in Ascomycota, at the phylum level, making a total of 4 taxonomic jumps (J = 4, species, genus, order, and family). The family nectriaceae contains other genera beyond *Fusarium* (i.e., *Nectria, Gibberella*), and thus we count at least one loss (L = 1). At the next level, order hypocreales, we have members that are in the database and are not part of nectriaceae (i.e., *Hypocrea, Cordyceps*), so we count an additional loss (L = 1 + 1 = 2). We repeat the process for the next taxonomic levels, reaching total of 4 losses (L = 4). Since J > 2 and L > 3, we assume that this may be a HGT event. **(C)** Abaccus performs a confirmation step by repeating the same procedure in a subsequent iteration repeating the process with the next sister branch (red dot). The next sister branch includes members of three genera in the family trichocomaceae, which again has as first shared taxonomic level phylum ascomycota. Now we have that J = 4 and L = 4, for which J ≥ 2 and L ≥ 3 is true. Having a second positive result implies that we accept the *F. oxysporium*, along with the sequence in *F. graminearum*, as an HGT event.

We constructed a custom python script (Abaccus) based on the ETE v2.3.10 (Huerta-Cepas et al., 2010) package that uses the previously described species taxonomy and the constructed gene trees to infer HGT events. This script is publicly available at https://github.com/Gabaldonlab/Abaccus and the version used here is v1.0. The Abaccus algorithm works as follows (see schematic example at Figure 1).

Given a tree we define the seed protein as the eukaryotic protein of interest. The taxonomic classification of a node is the lowest taxonomic category shared between all species included in a given node. For instance the taxonomic classification between *Fusarium oxysporum* and *F. graminearum* is genus (Fusarium) while the one between *F. oxysporum* and *Aspergillus nidulans* is at phylum (Ascomycota).

We first root the tree at the farthest leaf from the seed protein found in the tree. Then we run through every node from the seed protein to the root node. For each node we determine the taxonomic classification of the node and its parent node. Then we compare the two taxonomic classifications. The “jump” parameter (J) is defined as the difference between the taxonomic level found at the parent node and the one found at the current node. As seen in Figure 1A the jump parameter between *F. oxysporum* and *F. graminearum* is equal to 1 because we move from species level to genus level while in Figure 1B the jump parameter between *Fusarium* and *A. nidulans* is equal to four because we jump from genus level to phylum level. We then compute the minimal number of loss events (L) between the node and its sister node. We use a very parsimonious approach that infers that for each taxonomic level of difference between a node and its parental node one single loss event has happened only if there is at least a species in our database belonging to that taxonomic classification level that is not present in the nodes. This also implies that no loss is inferred if no other member of a given taxonomic category is present in the database. In this study we consider nodes that have a J ≥ 2 and L ≥ 3 as possible HGT events.

When both the taxonomic distance and minimal losses criteria are met (J ≥ 2, L ≥ 3), the program checks that both criteria are also met for the next sister branch (see Figure 1). This double check was used to limit the amount of false positives and to provide information of the taxonomic range for the putative donor. If this second condition is also true, the program retrieves the phylogenetic tree as a candidate for a HGT event and is selected for manual inspection. This consists of BLAST searches against the whole Uniprot database to ensure that the predicted scope of the events is coherent and does not disappear with additional data; that the detected homology is not spurious due to low identity percent saturating the phylogenetic signal; that the identity percent is neither too high, which may imply contaminating sequences in the primary genomic data rather than true HGT; and that the observed relationships are not due to fragmented or mispredicted genes. This step is performed manually because all these tasks would be difficult for a computer to handle and would require the application of arbitrary filters that would miss some events. The manual inspection also allow the detection of particular cases, such as species belonging to clades with reduced genomes, such as intracellular parasites, for which we expect higher gene loss rate. We assessed the accuracy of Abaccus following several criteria including (i) agreement with manual curation of predicted cases; (ii) ability to detect previously detected cases (Table 2), and (iii) agreement of the automated method with the manual inspection of the phylogenetic tree of the whole Asp_glu_race family. This tree contains dozens of independent eukaryotic clades, suggesting several independent HGT events into different lineages. Abaccus is able to identify most of the clades as putative HGT (Marcet-Houben and Gabaldón, 2010).

**Table 2:** **List of HGT events described in bibliography and detected in this study**.

**Uniprot ID**	**Species**	**Protein family**	**References**
68BA25	*Candida parapsilosis*	Pro_racemase	Fitzpatrick et al., 2008
O59828	*Schizosaccharomyces pombe*	Ala_racemase_N	Uo et al., 2001
K4DJR1	*Trypanosoma cruzi +*	Pro_racemase	Chamond et al., 2009
Q0UXH0	*Phaeosphaeria nodorum +*	Asp_glu_race	Marcet-Houben and Gabaldón, 2010
Q6FWC7	*Candida glabrata*	Asp_glu_race	Marcet-Houben and Gabaldón, 2010
Q0CB54	*Aspergillus terreus*	Asp_glu_race	Marcet-Houben and Gabaldón, 2010
Q0CB73	*Aspergillus terreus +*	Asp_glu_race	Marcet-Houben and Gabaldón, 2010
GORB82	*Hypocrea atroviridis +*	Asp_glu_race	Marcet-Houben and Gabaldón, 2010
F9FL25	*Fusarium oxysporum +*	Asp_glu_race	Marcet-Houben and Gabaldón, 2010

*The symbol “+” after the species name indicates that the HGT event affects several species*.

### Protein family gene tree

To gain a more detailed insight into the evolution of the aspartate-glutamate racemase family, we reconstructed a phylogenetic tree comprising all 2239 homologs identified by a profile-search in both proka_ref50 and euka_proteomes. We used MUSCLE v3.8 (Edgar, 2004) with default settings for the multiple sequence alignment, and we constructed the tree using FastTree with default settings (Price et al., 2010). Tree visualization was performed using the ETE v2.3.10 package (Huerta-Cepas et al., 2010).

### Signal peptide prediction analysis

In order to infer whether the HGT genes are expressed intracellularly or secreted we analyzed all the predicted genes using SignalP v 4.1 with default settings (Petersen et al., 2011).

### Heterologous expression, purification, and activity determination of CAGL0D01210g

For heterologous expression of the putative racemase gene from *C. glabrata* the complete coding sequence of CAGL0D01210g was selected (Marcet-Houben and Gabaldón, 2010). Its sequence has ~60% identity with aspartate racemase genes in *Lactobacillus* sp. and other bacteria; while no similar sequence can be found in other yeast species. Genomic DNA of strain ATCC2001 served as template and amplification was performed with Phusion polymerase (Thermo Fisher Scientific, Braunschweig, Germany) using oligonucleotides BamCglaRac_f (5′- GGA TCC ATG AAG GTT GGG ATT ATA GGT G -3′) and HindCglaRac_r (5′- AAG CTT CTA GCA AGA GAG TTT CTT TAC -3′) that add a *Bam*HI and *Hind*III restriction site to the respective ends of the amplicon. The fragment was cloned into the pJet1.2 vector using the CloneJET PCR Cloning Kit (Thermo Fisher Scientific) with subsequent transformation of *Escherichia coli* DH5α. Plasmid DNA was isolated with the NucleoSpin Plasmid isolation kit (Machery-Nagel, Düren, Germany) and checked by PCR with oligonucleotides ContCglaRac_f (5′- GTT CAT TGG ATG TTG CCA GTG -3′) and HindCglaRac_r. The gene was excised with *Bam*HI and *Hind*III and ligated into a *Bam*HI/*Hind*III restricted modified pET41.3 vector that adds an *N*-terminal His-tag to the protein (Hortschansky et al., 2007). After transformation of *E. coli* DH5α, plasmid DNA was isolated, sequenced and used for transformation of *E. coli* BL21 (DE3) Rosetta2 cells (Novagen/Merck). Expression was induced in 20 ml cultures of Overnight Express Instant TB medium (Novagen). Cells were harvested by centrifugation at an OD_550_ of 14, suspended in buffer A (50 mM Tris/HCL, 150 mM NaCl, 20 mM imidazole; pH 8.0) and disrupted by sonication. After 5 min centrifugation at 14 000 × g the cell-free extract was filtered over a 0.45 μm filter (Sartorius, Göttingen, Germany) and applied to a 1 ml gravity-flow Ni-Sepharose (GE Healthcare) column. The column was washed with 6 column volumes of buffer B (same as buffer A, but with 40 mM imidazole) and finally eluted with 5 ml of buffer C (same as A, but with 200 mM imidazole). Eluates were concentrated and desalted using centrifugal filter devices with a 10 kDa cut-off (Merck, Millipore). Analysis by SDS-PAGE (4–12% NuPage Bis-Tris gel; Thermo Fisher Scientific) and Coomassie staining revealed a purity of about 95%. d-Aspartate, d-alanine and d-glutamate were used in varying concentrations of up to 10 mM in 100 mM HEPES buffer pH 7.5 with up to 1 mg purified protein in 1 ml reactions to determine the time dependent change in optical rotation at 356 nm on a P-1020 polarimeter (Jasco, Gross-Umstadt, Germany). Alternatively, a coupled assay with d-amino acid oxidase as described previously was performed (Okada et al., 1991).

### Stress resistance and utilization of d-Amino acids by *C. glabrata* wild type strains and racemase mutants

The ORF CAGL0D01210g was deleted in the wild-type strain ATCC2001, and in the clinical isolate BAK618 (a kind gift from O. Bader, Göttingen), which is a *C. glabrata* wild-type strain of vaginal origin. The ORF was replaced with a nurseothricin resistance cassette (NAT1) containing a unique barcode and flanked by ≈500 bp homologous regions as described previously (Schwarzmüller et al., 2014). Correct integration and replacement of the original gene was confirmed by PCR. The CAGL0D01210g deletion mutants (two of each strain background) were grown on standard YPD agar plates at 30 and 42°C for testing heat stress resistance. In addition, YPD media were supplemented with 1.5 M NaCl for osmotic stress, 10 mM H_2_O_2_ for oxidative stress or 12 mM DTT to test for ER stress. Furthermore, cell wall stress resistance was analyzed by addition of either 1 mg/ml Congo red, 100 μg/ml Calcofluor white, 200 ng/ml Caspofungin or 3 mg/ml caffeine. All plates other than those for heat stress were incubated at 30°C. Tests were performed as drop dilution assays from a dense YPD overnight culture at 30°C, ranging from 10 to 10^5^ cells/spot. Plates were evaluated at 24 and 48 h. To test for d-amino acid utilization growth tests with either d- or l-amino acids as nitrogen source were performed. As basal media either *Candida* minimal medium without ammonium sulfate (Otzen et al., 2013) but with niacin supplementation (0.4 mg/l) to compensate for the niacin auxotrophy of *C. glabrata* (Domergue et al., 2005) or yeast carbon base (YCB, BD, Heidelberg, Germany) medium was used. Media were supplemented with 10 mM of either d- or l-alanine or d- or l-aspartate as nitrogen sources. Wells of a 96-well plate (Nunclon 96-well flat bottom plate, Thermo Fischer Scientific) were filled with 200 μl of the respective medium and seeded with 20,000 cells of the different wild-type and racemase mutant strains. Plates were sealed with gas permeable moisture barrier transparent sealing film (4 titude, Berlin, Germany) and growth was monitored using a microplate reader (Tecan infinite 200 pro, Tecan, Crailsheim, Germany) using the following settings: incubation temperature at 30°C; reading at OD_600_ with 3 × 3 reads per well, 10 s shaking before each reading, 15 min interval time for 97 cycles. Data were blank corrected against sterile control wells containing the respective media.

### Sequence analysis for CAGL0D01210g

Sequences corresponding to the *C. glabrata* gene CAGL0D01210g from 32 different isolates (F15, M17, P352, P353, B1012M, B1012S, BO101S, CST109, CST110, CST78, CST80, EB101M, F1019, F1822, F2229, I1718, M12, M6, M7, CBS138, BG2, CST34, CST35, E1114, EB0911Sto, EF0616Blo1, EF1237Blo1, EF1620Sto, EI1815Blo1, EG01004Sto, F03013, F11, and F15021) were retrieved. The sequences are provided as Supplementary File 1. DNASP v5 (Librado and Rozas, 2009) was used to compute genetic diversity at the synonymous and non-synonymous sites.

## Results

### A significant fraction of eukaryotic d-Amino acid metabolism is the result of HGT

From the literature we retrieved eight different families involved in d-amino acid metabolism (Table 1). We performed profile searches with their PFAM domains against Uniprot eukaryotic proteomes (See Section Materials and Methods), which resulted in 2165 proteins as putative members of the analyzed families present across 478 eukaryotic genomes. Each eukaryotic genome contains an average of 4.5 genes in the families analyzed. The number is highly variable (standard deviation of 5.9), ranging from organisms with no detected genes to others with dozens of them (*Macrophomina phaseolina* proteome has 41; *Hordeum vulgare* proteome has 40; mouse proteome has 33 and both *Magnaporthe oryzae* and *Glarea lozoyensis* have around 30 sequences).

We next assessed possible cases of HGT among these proteins. For this we used each eukaryotic d-amino acid metabolism protein as a seed in a pipeline that detected homologs across the diversity of sequenced organisms and performed phylogenetic reconstruction using a maximum likelihood approach (See Section Materials and Methods). Using Abaccus, a novel HGT detection algorithm described here which involves assessment of the tree topology in a taxonomic framework (See Section Materials and Methods), we identified a total of 121 proteins whose phylogenetic tree meets the established criteria (minimal number of implied parallel losses equal or greater than 3, and distance between taxonomic levels equal or greater than 2). We performed manual inspection of each of the 121 phylogenetic trees including a putative HGT event. After manual inspection and rejection of 14 events, we identified a total of 48 possible independent HGT events. Each of the events resulted in 1–33 acquired genes through transfer followed by lineage diversification and gene duplication. The 48 events contain a total of 223 proteins affecting 116 eukaryotic species analyzed (Supplementary Table 2). Of these proteins, 73 were not detected by the initial HMM-based survey used to predict the global d-amino acid metabolism proteome, and thus the remaining 150 proteins (223 proteins—73 non-detected proteins)—represent 7.0% of the eukaryotic d-amino acid metabolism proteome. Given our highly stringent threshold, this should be considered a minimal estimate. Since the pipeline depends on representation of at least an additional member of each taxonomic category between the conflicting lineage and its sister branch, we expect it to be less sensitive when applied to poorly sampled groups or organisms that branch in very basal position compared to other members of the same group. For instance, the placozoa *Trichoplax adhaerens* is the only known member of its phylum, that also is a basal metazoa. These characteristics makes it a very poor candidate for the detection of HGT in its genome by Abaccus. Several of the main eukaryotic lineages have a very small number of sequenced representatives, such as amoebozoa, rhizaria, or hacrobia; while some others are currently represented in databases mostly by extreme parasites, such as excavata. For all these groups the requirement of the minimal number of losses can hardly be met and thus we expect Abaccus to be missing almost any potential HGT event in those clades.

### Global patterns of horizontal gene transfer of d-Amino acid metabolism across eukaryotes

**Figure 2:**
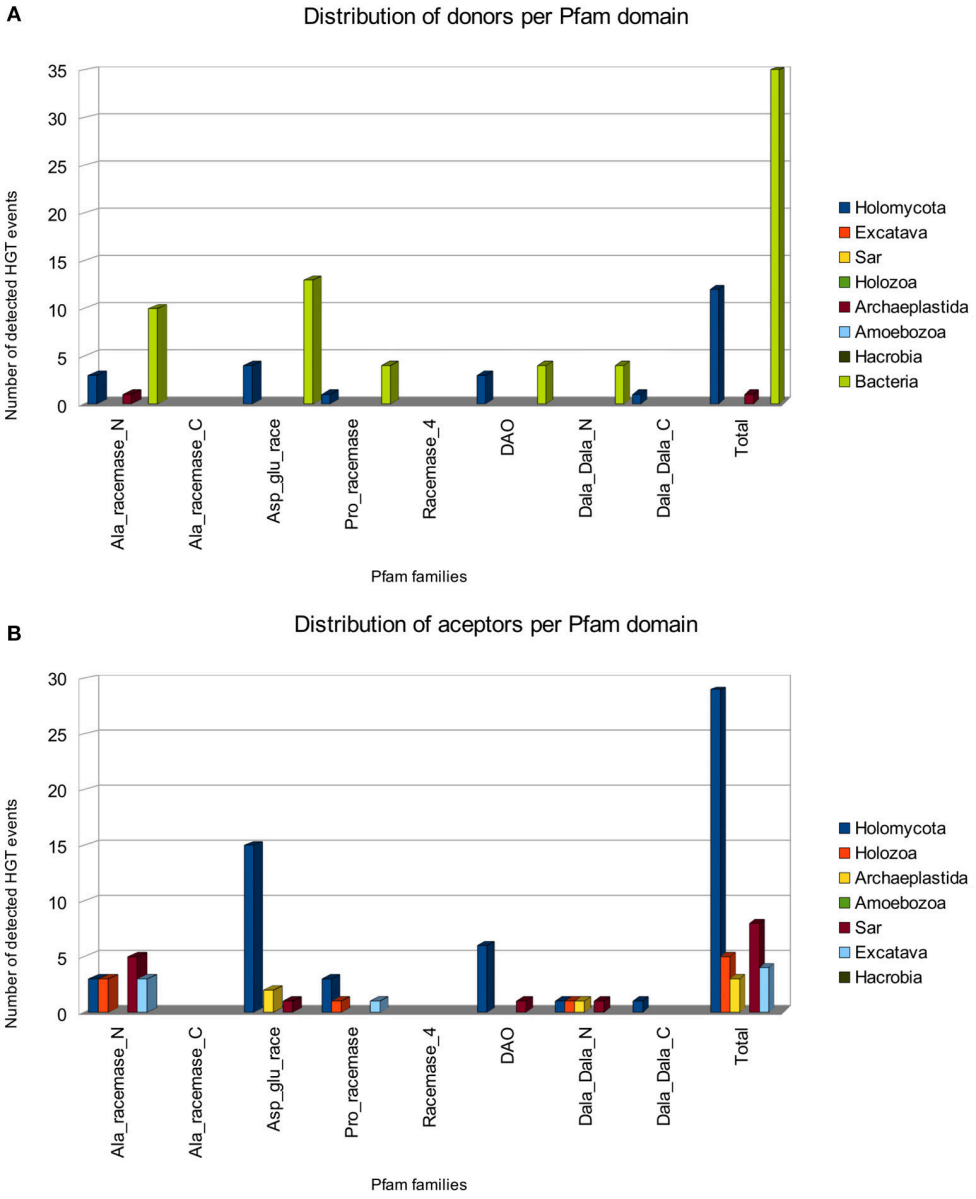
**Distribution of HGT events across the different eukaryotic superkingdoms**. Distribution of number of transferred proteins per family is shown as bar plots in which the taxonomic distribution of donors **(A)** and acceptors **(B)** are indicated with different colors.

**Figure 3:**
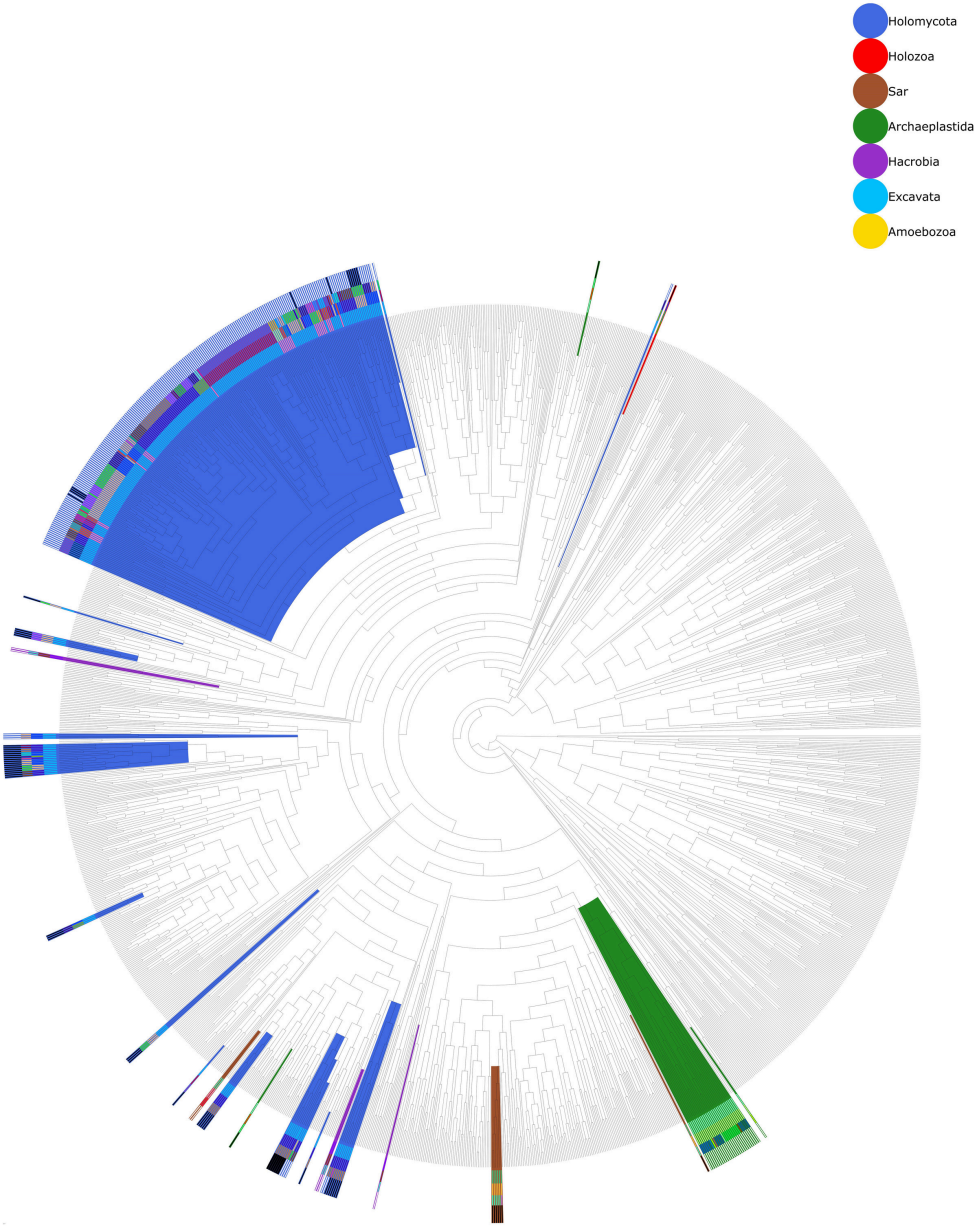
**Phylogenetic tree of the whole Asp_glu_race family**. Background colors indicate branches containing sequences from one eukaryotic superkingdom. Each leaf was assigned a four color code. The inner band correspond to a color for each phylum (i.e., Ascomycota). The next two bands are random colors assigned for the categories of class and order. The most external band has only two values, black and white, and indicate whether the sequence has been detected as participant in an HGT event using Abaccus (Black = True; White = False).

The distribution of HGT events across enzyme families and taxonomy of putative donor and acceptor clades is shown in Figure 2. Interestingly, more than one third of the events (17 events, 34.7%) correspond to the aspartate-glutamate-hydantoin racemase family (Asp_Glu_race). This family encodes enzymes acting on chiral centers in acidic amino acids (glutamate and aspartate) and several other nitrogenated compounds that share a common backbone, such as hydantoin or aryl malonate (Abe et al., 2005; Friedman and Island, 2010). Fifteen out of seventeen events have fungi as acceptors for the transferred gene. The other two have acceptors in archaeplastida (*Physcomitrella patens*) and sar (oomycota). We found that in 13 out of 17 HGT events the putative donor group lies within Bacteria and in four the putative donor lies within fungi. However, at least three events (bacteria to fungi) involve several phylogenetically unrelated fungal genera, and a fourth event (HGT between fungi) is embedded within another detected event (bacteria to fungi HGT). All this suggests a nested scenario with bacterial genes transferred to a fungal lineage and subsequent dissemination of the gene via HGT between different fungal lineages. Also, of the 15 events with fungal acceptors, 13 involve one or more plant pathogenic species (Supplementary Table 3); and there is another event involving plant pathogens from the oomycetes. However, in this case the event seems to predate the split between the plant pathogenic Peronosporales and the fish pathogens of Saprolegniales. While it can be argued that plant pathogenic fungi are overrepresented in current databases due to their economical relevance, many of the predicted HGT events present a taxonomic distribution that is expected to include non-plant-associated and phylogenetically close relatives. In these cases, either the gene was present in the common ancestor of the observed plant-associated fungi and its relatives where it was lost, or the observed distribution is totally or partially due to HGT affecting preferentially plant-associated fungi. In any case, our results suggest a trend for either favoring the loss or preventing the acquisition of d-amino acid metabolic genes in species without plant-associated lifestyles, or the acquisition by plant-associated organisms. We reconstructed the complete phylogenetic tree of the Asp_Glu_race family (Figure 3) to obtain a clearer picture of the evolutionary events involved. The tree revealed the existence of more than 20 independent and unrelated eukaryotic lineages of Asp_glu_race proteins; most of them showing a phylogenetic pattern that deviates greatly from the species phylogeny. Furthermore, many of the groups of eukaryotic enzymes have a very patchy phylogenetic distribution, and we can observe several unrelated clades that include otherwise highly related organisms.

The second most commonly transferred family is Ala_racemase_N, with 14 events. This family encodes pyridoxal phosphate-dependent enzymes typically acting on chiral centers of amino acids. For this family, the most common acceptor taxonomic group is the SAR supergroup of microbial eukaryotes (stramenopiles, alveolates, and rhizaria), with five events. These include organisms as diverse and with distinct lifestyles such as *Paramecium, Cryptosporidium*, the oomycetes, and the bacillariophytes; thus, there is no apparent ecological or physiological connection among them. Its followed by holozoa and excavata, both with three events and holomycota, with two events. One of the more unexpected events in this family is a putative transference from chlorophyta to the parasitic trypanosomids *Trypanosoma* and *Leishmania*. Blast against Uniprot retrieves hits in other related parasitic genera (*Strigomonas, Angomonas, Phytomonas*), but no hit can be found in related free-living organisms, such as bodonids. We suspect this may be due to absence of sequences of these organisms in the databases.

Third in number of events is the DAO family (d-amino acid oxidase), which comprises enzymes catalyzing the degradative oxidation of d-amino acids. The seven detected events are spread across fungi (6), and oomycetes (1). In this family, four events have Bacteria as putative donor, and three fungi. Three of the events seem to contain secondary HGT, and all the cases with donor mapped into fungi have other fungi as acceptor group. This suggests that, as previously described for Asp_glu_race, bacterial DAO enzymes in fungal genomes are commonly transferred to other, unrelated fungi. Even more, just like in the previous case, all the events with fungi as acceptor involve one or more species of plant pathogens; and another event can be found at the base of the oomycota.

Regarding the rest of the families, the number of detected events is lower and no apparent patterns arise. All the events found for Ala_racemase_C are detected when analyzing Ala_racemase_N (many enzymes in the family, but not all, contain both domains). For Pro_racemase we detected a protein in *Nematostella vectensis* with 85% identity with *Pseudomonas* sp. Such identity value would imply either an extremely recent event or a contamination. Given that *Pseudomonas* is a very ubiquitous genus and that the probability of HGT in metazoans is expected to be rather low, we prefer to treat these events with caution until further analyses provide more robust evidence. Another event in the family comprise a putative hydroxyproline racemase in *Pseudogymnoascus destructans*. The gene shares around 75% identity with sequences in alphaproteobacteria, although the blast alignment covers only a portion of the gene. We suspect the gene may have a mispredicted end. The other two events: proline racemase in several species of *Trypanosoma* sp. (Chamond et al., 2009) and in *Candida parapsilosis* (Fitzpatrick et al., 2008) were previously described in the literature. Dala_Dala_N encodes the binding domain of several enzymes that act on the d-alanine dimer that forms part of bacterial peptidoglycan. Four events are found for this family. One of them has *Physcomitrella patens* as apparent acceptor, but the identity values (86%), and the fact that other events with similar characteristics are identified for this species suggests that the presence of a high number of contaminating sequences in the proteome is more likely than a very recent boom in gene acquisition. The rest of the events are a protein in the nematode *Caenorhabditis remanei*, with 60% identity with actinobacterial sequences; a protein in the heterokont algae *Aureococcus anophaggeferens*, with ≈35% identity with other bacterial sequences (may be a rather ancient event); and a gene that seems to have been transferred from bacteria to the base of the Sordariaceae family, being present in *Sordaria* and *Neurospora*, and with a 45% identity to sequences coming from the bacterial phyllum bacteroidetes. The only unique event in the Dala_Dala_C domain is a fungi to fungi transfer from eurotiomycetes fungi to *Colletotrichum gloeosporoides*; another plant pathogen. We detected no event affecting the Racemase_4 family.

**Figure 4:**
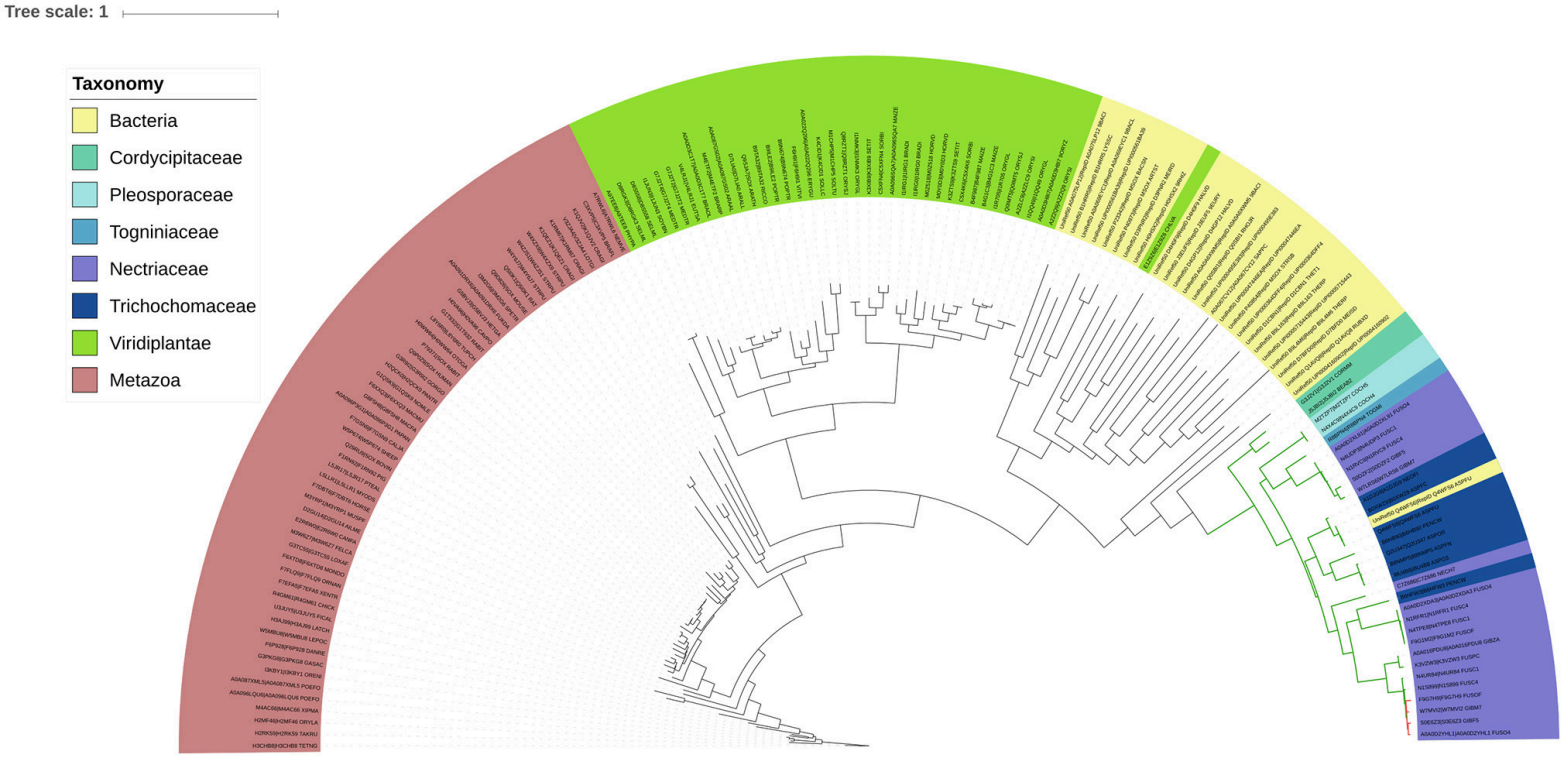
**Phylogenetic tree of a detected HGT event in the family of DAO**. The color code indicates the phylogenetic affiliation of the different sequences. Branches colored in green indicate the clade corresponding to the predicted HGT event. Branches colored in red indicate the presence of a predicted signal peptide. Regarding the phylogenetic affiliation of the represented sequences it is noteworthy that the fungal sequences correspond either to the Sordariomycetes (Cordycipitaceae, Pleosporaceae, and Nectriaceae are members of the Hypocreales; while Togniniaceae is part of Togniniales); or Eurotiomycetes (Trichochomaceae in the order Eurotiales). Regarding this second group, their position in the tree is incongruent with the species phylogeny and may indicate secondary HGT. Sequences colored as yellow correspond to Uniref50 clusters of prokaryotic sequences. The rest of the tree is composed by metazoan and plant sequences, colored in red and bright green, respectively. The tree was visualized using iTOL web tool (Letunic and Bork, 2016).

All in all, as seen in Figure 2B we observed that bacteria are the most common donor, followed by fungi. Bacteria to fungi is the most common type of events (19 cases), followed by fungi to fungi (10 cases), bacteria to SAR (6 cases), and bacteria to metazoa (5 cases) (Table 3). With respects to the different families involved in racemase metabolism, three of them (aspartate-glutamate racemase, alanine racemase, and DAO) appear to be the ones that are more often transferred. DAO contains the only instance we have found of an HGT gene encoding a protein with a predicted signal peptide. Curiously, it corresponds to a monophyletic subgroup of genes in four members of the family Nectriaceae within a wider event that involve other fungi both in the same and other families (Figure 4). The four species present two genes within the event, one with predicted signal peptide and the other without it; although some other members of nectriaceae also have two genes in the same event. All of this suggests that the HGT acquired gene has been duplicated in this fungal family, followed by neofunctionalization in a subgroup that has apparently retargeted one of the gene products for an extracellular function.

**Table 3:** **Donor-aceptor pairing**.

	**Holomycota (Acceptor)**	**Holozoa (Acceptor)**	**Archaeplastida (Acceptor)**	**Amoebozoa (Acceptor)**	**SAR (Acceptor)**	**Excavata (Acceptor)**	**Hacrobia (Acceptor)**
Bacteria (Donor)	18	5	2	0	6	3	0
Holomycota (Donor)	10	0	0	0	2	0	0
Holozoa (Donor)	0	0	0	0	0	0	0
Archaepl. (Donor)	0	0	0	0	0	1	0
Amoebozoa (Donor)	0	0	0	0	0	0	0
Sar (Donor)	0	0	0	0	0	0	0
Excavata (Donor)	0	0	0	0	0	0	0
Hacrobia (Donor)	0	0	0	0	0	0	0
Total	28	5	2	0	8	4	0

*The table indicates the number of HGT events for each acceptor/donor pair*.

### No obvious role of bacterial-derived aspartate glutamate racemase in *Candida glabrata*

In order to investigate possible physiological roles of the transferred racemases we selected the transferred aspartate glutamate racemase in *C. glabrata*, encoded by the gene CAGL0D01210g. This event was described previously (Marcet-Houben and Gabaldón, 2010) and detected again in the present study. *C. glabrata* is an opportunistic pathogenic yeast which is evolutionarily closely related to the model organism *Saccharomyces cerevisiae* (Gabaldón and Carreté, 2016). It is therefore well-suited for genetic manipulation and laboratory experimentation using tools developed for *S. cerevisiae*. The gene had been knocked out in an earlier study as part of a systematic deletion of *C. glabrata* ORFs in an ATCC2001 *his3*Δ/*leu2*Δ/*trp1*Δ genetic background (Schwarzmüller et al., 2014). In that study the mutant presented no phenotype in any of the conditions tested which included the ability to form biofilms, resistance to anti-fungal drugs (azole and amphotericin B), and cell-wall, heat and osmotic stresses. In addition it presented no apparent growth defects or anomalous colony morphology, and did not present significant growth defects in standard medium as compared to the wild type. A subsequent study included that mutant in a virulence screening using a *Drosophila* infection model (Brunke et al., 2015). The deletion mutant presented no significant difference in virulence compared to the wild type strain. Analysis of the genome sequence of 32 distinct *C. glabrata* clinical isolates, however, shows the presence of the gene in all of the strains and a one-fold excess of ratios of synonymous (Πs 0.034) to non-synonymous polymorphisms (Πn 0.0036, See Section Materials and Methods). Therefore, the transferred gene seems to be under purifying selection at the species level and a biological function is expected.

In an attempt to further characterize this gene, we constructed deletion mutants (See Section Materials and Methods) of the same gene in two alternative genetic backgrounds and tested the phenotype upon heat, osmotic, oxidative, ER, and cell wall stresses, which revealed no phenotypic difference between the mutants and the corresponding wild-type strains. We next tested the growth of the mutant and wild type strains in media containing l- or d- alanine and aspartate amino acids. While the l-form of the amino acids alanine and aspartate served as nitrogen sources for *C. glabrata* wild-type and mutant strains, none of the respective d-amino acids served as nitrogen source (Supplementary Figure 1). No difference in growth between wild type and respective mutant was observed (Supplementary Figure 2). We finally produced the *C. glabrata* racemase as a soluble protein in *E. coli* and purified it to >95% purity by Ni-chelate chromatography (See Section Materials and Methods) to be used in enzymatic assays. No change in optical rotation using either d- or l-alanine or d- or l-aspartate or d- or l-glutamate was observed. In addition, coupled assays using d-amino acid oxidases remained negative. Altogether, these results suggest that the transferred racemase in *C. glabrata* may not be involved in d-aspartate or d-glutamate utilization.

## Discussion

Our phylogenetics approach allows the efficient detection of highly likely HGT events provided there is a sufficient broad taxonomic coverage of the sequenced genomes, such as in fungi. In fact, we were able to detect several cases of HGT between more or less distantly related fungal groups. In this regard, we consider that our analysis demonstrate that: (i) Enzymes similar to proteins related to d-amino acid metabolism have been horizontally transferred several independent times from bacteria to fungi, supporting previous observations (Marcet-Houben and Gabaldón, 2010); (ii) many of these events involve a transfer from bacteria to fungi, followed by subsequent transferences between fungi; (iii) fungi to fungi HGT events seem to be rather common, with an apparent number of events comparable in magnitude to bacteria-to-fungi HGT, at least for some protein families; and (iv) plant-associated fungi seem to be more affected by those transferences than any other eukaryotic group, with several independent events in many unrelated and ecologically very dissimilar organisms (Fisher test *p*-value = 3.4e^−13^). This implies that either HGT in general, or HGT of some particular families such as aspartate-glutamate-hydantoin racemases, are more common in plant associated fungi than in fungi inhabiting other niches; or that fungi with a well-developed d-amino acid metabolism have an advantage in plant-associated environments. We argue that, since some of the events seem to have occurred at the base of some taxa containing both plant pathogens and non-plant pathogens, with aparent secondary loss in non-plant pathogens; the hypothesis of a higher prevalence of HGT in these organisms is unlikely. Thus, it is more likely that d-amino acid metabolism can provide an advantage in those conditions and be lost otherwise. Acidic d-amino acids are fairly abundant in environments with high bacterial activity or high rates of spontaneous racemization (due, for instance, to high radiation exposure, high temperatures or extreme pH; Vranova et al., 2011; Steen et al., 2013). This means that, under the right conditions, d-amino acids are fairly abundant and perhaps constitute an underexploited nitrogen source. Many fungi can degrade cellulose, hemicellulose or pectin to simple monosaccarides, and many species in the subphylum agaricomycotina can even feed on lignin (Dashtban et al., 2010; Floudas et al., 2012; Sigoillot et al., 2012). Fatty acids are abundant in some plant structures, such as seeds, and can also be used as carbon source for many fungi (Wilson et al., 2004; Wang et al., 2007). Thus, plant biomass is an easily exploitable carbon source for fungi. Compared with the high availability of carbon in plant tissues, nitrogen is rather scarce in living plants and even rarer in plant necromass due to nutrient translocation during senescence (Hörtensteiner and Feller, 2002; Avila-Ospina et al., 2014) and by competing microbes. Given this scenario, the ability to take advantage of rarer nitrogen sources should provide a selective advantage, as predicted by the stoichiometric theory of microbial ecology (Hartman and Richardson, 2013; Waring et al., 2013; Chen et al., 2014; Mooshammer et al., 2014). This implies that genes related to the exploitation of other uncommon nitrogen sources should be more commonly duplicated or transferred in microorganisms that are well-adapted to grow on living or dead plants. This includes plant pathogens, but also other related niches such as wood-decaying fungi or endophytic fungi. An implication of this hypothesis is that, for plant pathogens, mutants defective for these d-amino acid related metabolic enzymes probably will not lose their pathogenicity. Such mutants may be out-competed during co-infection with wild strains, probably in a manner that depends on abiotic factors (for instance soil type, temperature or luminosity). This hypothesis also implies that probably those same gene families play a similar role in other plant-feeding microbes, such as certain oomycetes. In this regard, we have identified two events that happened at the base of oomycetes in both the Asp_glu_race and DAO families. Unfortunately, oomycetes genomics is not as well-developed as fungal genomics, with a taxonomic sampling that represents only a small fraction of the described biodiversity.

An additional explanation for such prevalence in HGT for the enzymes for d-amino acid metabolism could be its involvement in the synthesis of products of non-ribosomal peptide synthetases and hybrid polyketide synthetases. While the genes involved in this kind of secondary metabolites are expected to be easy to be transferred horizontally due to their peripheral placement in metabolic networks and having high potential phenotypic effects even at low expression levels; the architecture of these proteins makes them very difficult to detect with the methodology of this study. In eukaryotes, NRPS and PKS usually form very long proteins with repeated structural motifs formed by regular concatenation of protein domains. Each structural motif adds a single component to the final polymeric product. Racemase activity is usually found in some accessory domains of these proteins. However, these genes have regions with high variability and suffer from frequent domain rearrangements, which makes them unsuitable for traditional phylogenetic reconstruction. None of the identified events include proteins with any of the non-ribosomal peptide synthetase or polyketide syntethase standard protein domains, and although some of the events may be in fact functionally linked to any of these proteins, perhaps as part of a metabolic cluster, it seems unlikely that this hypothetical circumstance could explain the whole trend.

Our experimental results indicate that d-amino acids are likely not the substrate of the transferred racemase in *C. glabrata*. Although no apparent phenotype can be found for the null mutant, the sequence conservation of the transferred gene in all *C. glabrata* strains analyzed suggest it indeed has a function. This highlights the need to perform functional studies of horizontally acquired genes, as their function may have been altered during evolution. It also suggests that functions other than d-amino acid degradation should also be considered as potential selective advantage of the acquisition. Of note *C. glabrata* is a human commensal and an opportunistic pathogen and, although some of its relatives can be isolated from plant parts such as flowers and fruits (Gabaldón and Carreté, 2016), this clade does not present the ability to degrade plant structural polymers. Further research is needed to test the hypothesis put forward here to explain the increased occurrence of prokaryotic racemases in plant-associated fungi as well as to clarify the elusive function of the prokaryotic racemase acquired in *C. glabrata*.

## Author contributions

MN, MM, TG conceived the project and planned the computational approach. MN performed the computational analyses and wrote the Abaccus software. MN and TG designed the Abaccus algorithm. MB, SB, and BH designed and performed the experiments on *C. glabrata*. MN and TG wrote the first draft of the manuscript. All authors contributed to the final version of the manuscript.

### Conflict of interest statement

The authors declare that the research was conducted in the absence of any commercial or financial relationships that could be construed as a potential conflict of interest.

## Supplementary material

Supplementary Figure 1 **Growth of *C. glabrata* wild types on solid media with d- and l-amino acids**. The sequenced wild type strain ATCC2001 and the clinical isolate BAK618 grow on glucose-containing medium with l-alanine or l-aspartate as nitrogen source, but not with their d-enantiomers. In comparison, the *C. albicans* wild type SC5314 containing d-amino acid oxidases can use all amino acids as nitrogen source, albeit with different efficiencies.

Supplementary Figure 2 **Growth of *C. glabrata* wild type and racemase mutant strains in liquid cultures**. The *C. glabrata* wild type ATCC2001 and the clinical isolate BAK618 grow in liquid minimal media (CMM or YCB), as long as an l-amino acid (alanine or aspartate, green symbols) serves as nitrogen source. d-alanine or d-aspartate (red symbols) do not support growth of any strain, and deletion of the transferred racemase gene CAGL0D01210g (Δ*rac1*) in either background has no influence on growth of *C. glabrata* with either enantiomer.

Supplementary Table 1 **List of the analyzed eukaryotic proteomes**.

Supplementary Table 2 **List of detected HGT events**. Events marked in red were rejected after manual inspection either because of high suspicion of contamination or due to phylogenetic incongruences with the result of a BLAST search against Uniprot database.

Supplementary Table 3 **List of plant-associated fungi and oomycota**. The table indicates if the species is considered plant-associated (Y) or not (N). Classification into one lifestyle or the other is based on wikipedia articles of the given species or bibliographical evidence. In the second case, citation is provided.

Supplementary File 1 **Nucleotide sequences of the Asp_Glu Racemase in the 32 *C. glabrata* strains analyzed (FASTA format)**.
