# Supplementary figures and images for: Widespread Inter- and Intra-Domain Horizontal Gene Transfer of d-Amino Acid Metabolism Enzymes in Eukaryotes
Source: Front Microbiol. 2016 Dec 20;7:2001. doi: 10.3389/fmicb.2016.02001 (PMC5169069; doi:10.3389/fmicb.2016.02001)

## Yeast carbon base with different N-sources

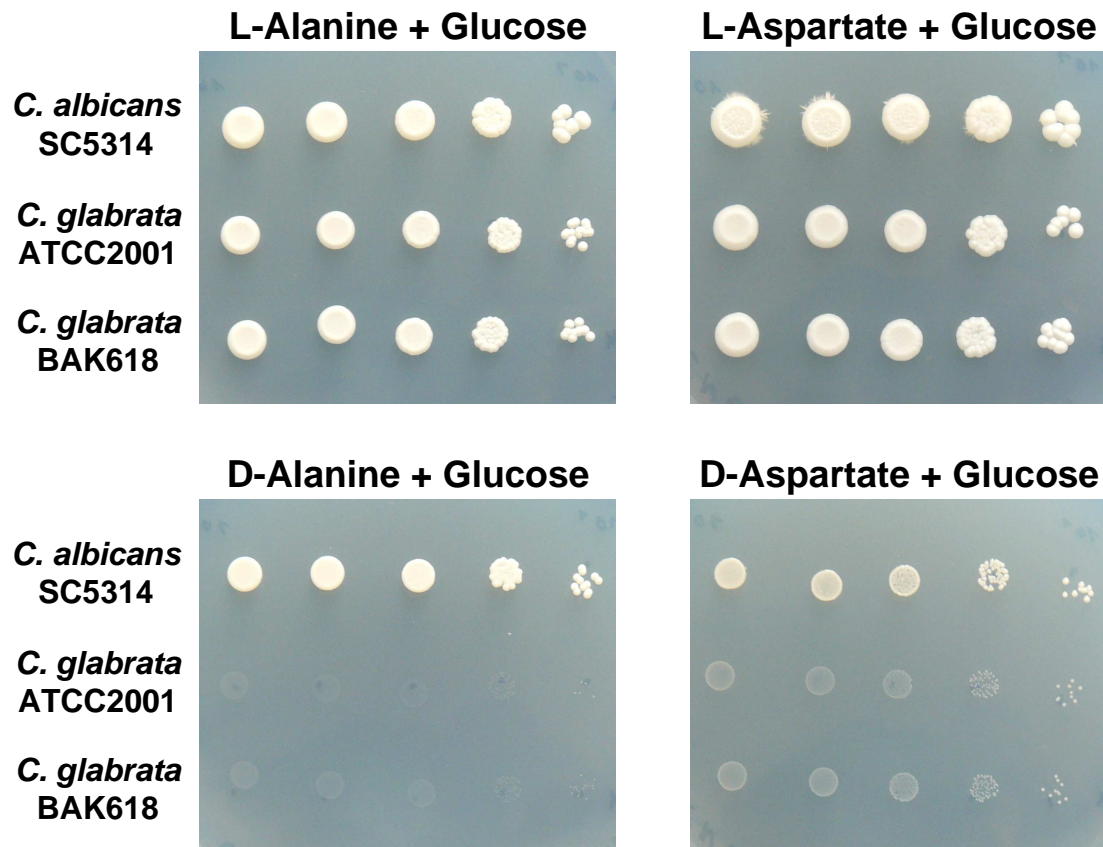

Supplement: Supplementary Figure 1 — Growth of C. glabrata wild types on solid media with d- and l-amino acids. The sequenced wild type strain ATCC2001 and the clinical isolate BAK618 grow on glucose-containing medium with l-alanine or l-aspartate as nitrogen source, but not with their d-enantiomers. In comparison, the C. albicans wild type SC5314 containing d-amino acid oxidases can use all amino acids as nitrogen source, albeit with different efficiencies. [file Image1.PDF]
