# Supplementary material for: Widespread Inter- and Intra-Domain Horizontal Gene Transfer of d-Amino Acid Metabolism Enzymes in Eukaryotes
Source: Front Microbiol. 2016 Dec 20;7:2001. doi: 10.3389/fmicb.2016.02001 (PMC5169069; doi:10.3389/fmicb.2016.02001)
Supplement: Supplementary Figure 2 — Growth of C. glabrata wild type and racemase mutant strains in liquid cultures. The C. glabrata wild type ATCC2001 and the clinical isolate BAK618 grow in liquid minimal media (CMM or YCB), as long as an l-amino acid (alanine or aspartate, green symbols) serves as nitrogen source. d-alanine or d-aspartate (red symbols) do not support growth of any strain, and deletion of the transferred racemase gene CAGL0D01210g (Δrac1) in either background has no influence on growth of C. glabrata with either enantiomer. [file Image2.PDF]

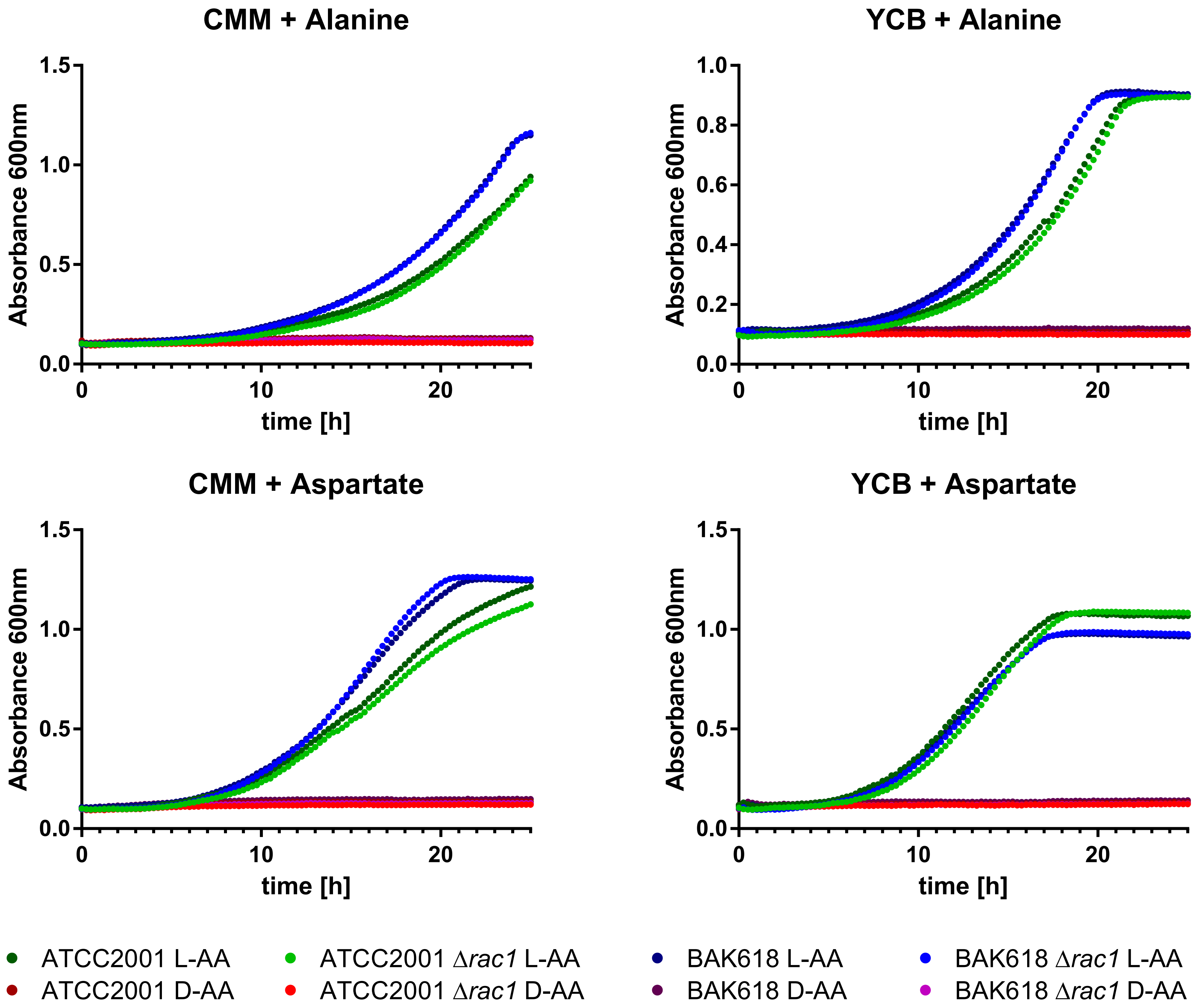
CMM + Alanine
YCB + Alanine
CMM + Aspartate
YCB + Aspartate
Absorbance 600nm
time [h]
0.0
0.5
1.0
1.5
0.2
0.4
0.6
0.8
0
10
20
ATCC2001 L-AA
ATCC2001 D-AA
ATCC2001 Δrac1 L-AA
ATCC2001 Δrac1 D-AA
BAK618 L-AA
BAK618 D-AA
BAK618 Δrac1 L-AA
BAK618 Δrac1 D-AA
